# Total Intravenous Anesthesia Maintained the Degree of Pre-Existing Mitral Regurgitation Better than Isoflurane Anesthesia in Cardiac Surgery: A Randomized Controlled Trial

**DOI:** 10.3390/jcm8081104

**Published:** 2019-07-25

**Authors:** Jin Hee Ahn, Hyun Joo Ahn, Jae-Woo Yi

**Affiliations:** 1 Department of Anesthesiology and Pain Medicine, Kangbuk Samsung Hospital, Sungkyunkwan University, School of Medicine, Seoul 03181, Korea; 2 Department of Anesthesiology and Pain Medicine, College of Medicine, Graduate School, Kyung Hee University, Seoul 02447, Korea; 3 Department of Anesthesiology and Pain Medicine, Samsung Medical Center, Sungkyunkwan University School of Medicine, Seoul 06351, Korea; 4 Department of Anesthesiology and Pain Medicine, College of Medicine, Kyung Hee University, Seoul 02447, Korea

**Keywords:** anesthesia, mitral valve insufficiency, isoflurane, propofol

## Abstract

Accurate assessment of mitral regurgitation (MR) is critical during mitral valve repair surgery. However, anesthesia may influence the degree of mitral regurgitation by changing pre- and after-load or cardiac contractility. Therefore, we compared changes in mitral regurgitation by total intravenous anesthesia (TIVA) and inhalation anesthesia in patients with pre-existing mitral regurgitation. This was a double-blind randomized controlled study conducted at a tertiary care center in 2018. Fifty-four mitral regurgitation patents undergoing elective cardiac surgery were randomly assigned to receive TIVA or isoflurane. Primary endpoint was change of regurgitation volume by anesthesia. The reduction of regurgitation volume by anesthesia was greater in the isoflurane group than in the TIVA group (mean (95% confidence interval CI): −0.20 (−6.15, 5.75) vs. −9.66 (−15.77, −3.56), mL·beat^−1^, *p* = 0.0266) and this phenomenon was more prominent with severe mitral regurgitation (grade 3 or 4) (mean (95% CI): −0.33 (−9.10, 8.44) vs. −16.20 (−24.22, −8.18), mL·beat^−1^, *p* = 0.0079). Among patients with MR grade 3 or 4, 94% remained the same with TIVA during anesthesia compared to 56% with isoflurane. In conclusion, TIVA maintained the pre-anesthetic state of mitral regurgitation relatively well, while the severity of mitral regurgitation tended to decrease with isoflurane anesthesia.

## 1. Introduction

Evaluation of the degree of mitral regurgitation (MR) is essential during anesthesia for mitral valve surgery [1]. If MR is found after cardiopulmonary bypass weaning, the decision must be made whether additional surgery is required or simple observation will do [1]. Hari et al. [1] demonstrated that late event free survival was significantly lower in patients with remnant MR after cardiac surgery.

However, the degree of MR may change during anesthesia. During general anesthesia, the overall sympathetic tone decreases, myocardial contraction decreases, and pre- and after-load decrease. The net effect can influence pre-existing MR and may result in measurement values different from the pre-operative assessment [2,3].

Inhalation anesthesia and total intravenous anesthesia (TIVA) are used alternatively for cardiac anesthesia. Inhalation agents are known to be effective for reducing perioperative myocardial ischemia-reperfusion injury with an ischemic preconditioning effect [4,5,6,7,8]. Most inhalation anesthetics decrease myocardial function, but cardiac output (CO) is relatively maintained due to a preserved baroreflex [9,10]. TIVA using continuous infusion of propofol and remifentanil offers the benefit of no air pollution, a short half-life and rapid recovery. Propofol, the key agent in TIVA, reduces cardiac contractility, but the ejection fraction is maintained with the vasodilation effect [11,12].

There have been few studies dealing with changes in pre-existing MR with different anesthetic agents. Consideration of anesthesia and the possibility of different effects of each anesthetic may be required before assessing the degree of MR and determining the need for further surgery [13], which has ramifications for postoperative outcomes. Therefore, in the current study, we investigated how much influence anesthetics have on the degree of MR, and which anesthetic better reflects the pre-anesthesia state of MR in patients undergoing cardiac surgery.

## 2. Materials and Methods

This prospective, randomized, parallel group study was approved by our Institutional Review Board (Ethical committee of Samsung Medical Center Institutional Review Board SMC 2017-07-107, Chairperson Prof. Suk-Koo Lee, date of approval Sep 09, 2017, Seoul, Korea) and registered prior to patient enrollment at the Clinical Research Information Service (KCT 0002767; Principle investigator: H J A; date of registration March 14, 2018, https://cris.nih.go.kr/cris). Written informed consent was obtained from all participants. This manuscript adheres to the CONSORT guidelines.

### 2.1. Patients

**Figure 1:**
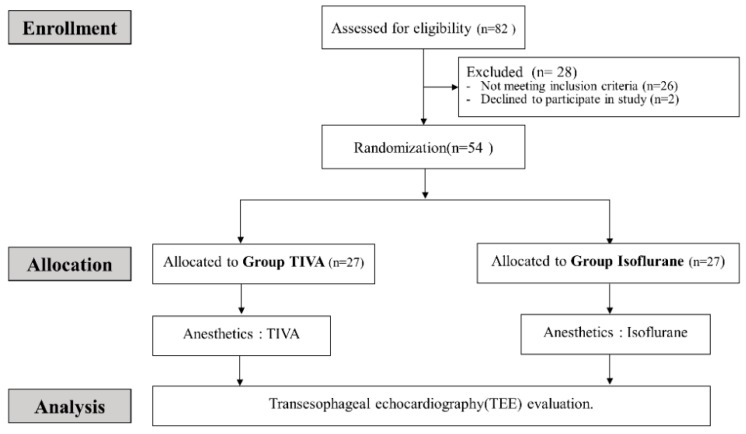
CONSORT flow diagram.

This study was performed between March and Oct 2018 at the Samsung Medical Center (Seoul, Korea). A total of 82 patients undergoing cardiac surgery were assessed for eligibility. Of these patients, 56 met the inclusion and exclusion criteria. Two patients declined to participate. Therefore, 54 patients were enrolled by study staff (Figure 1).

The inclusion criteria were patients undergoing elective cardiac surgery and having MR greater than mild, age > 19 years, American Society of Anesthesiologists physical status (ASA) I–IV. The exclusion criteria were: (1) absence of preoperative transesophageal echocardiography (TEE); (2) previous valvular surgery; (3) regional wall motion abnormality of akinesia, dyskinesia, or aneurysmal change at any segment; (4) Eigenmenger syndrome; (5) disease or surgical history in the gastrointestinal system that contraindicated insertion of TEE; and (6) coagulation abnormalities. Dropout criteria were measurement error, unstable hemodynamics, or inotrope/vasopressor administration during TEE measurement.

### 2.2. Randomization and Blinding

Randomization to receive TIVA or isoflurane was done by a computer-generated random numbers table (www.randomizer.org) and permuted-block randomization with a fixed block size. The sequential random numbers were concealed in opaque envelopes that were identical. An attending anesthesiologist who was not involved in the study opened the sealed envelope just before anesthesia induction and provided the allocated anesthetic drugs according to group assignment. A single designated anesthesiologist performed intraoperative TEE. During the TEE examination, the vital sign monitor, gas vaporizer and syringe pump were hidden with an opaque screen so that the anesthesiologist performing TEE did not know the type of anesthesia. The attending anesthesiologist recorded intraoperative hemodynamic variables. All views and measurements were stored in a picture archival and communication system (PACS, Centricity Enterprise Web V3.0, GE Healthcare, Palo Alto, CA, USA). All results were collected after the study was finished. The surgeon, patients, and data analyzer were blinded to group assignments.

### 2.3. Anesthesia Protocol

Anesthesia was maintained with propofol (80 to 120 mcg/kg/min) in the TIVA group and around 1 minimum alveolar concentration (MAC) isoflurane in the isoflurane group. Propofol or isoflurane was titrated for a bispectral index between 40 to 50. Remifentanil was continuously infused in both groups (0.0–0.2 mcg/kg/min). For muscle relaxation, 0.8 mg/kg rocuronium was administered before intubation and vecuronium was continuously infused for maintenance in both groups.

After endotracheal intubation, lungs were mechanically ventilated using tidal volumes of 6–8 mL/kg of ideal body weight, an inspired oxygen fraction of 0.5, and positive end-expiratory pressure of 5 cmH_2_O. The respiratory rate was adjusted to maintain end-tidal carbon dioxide at 35 to 40 mmHg.

### 2.4. TEE Measurement

#### 2.4.1. Preoperative TEE

Baseline TEE was performed by a cardiologist in the echocardiography laboratory. Patients fasted for at least 8 h before TEE examination, and Xylocaine (Aspen, Xylocaine^®^ 10% Pump spray, Luton, Ireland) was sprayed on the pharynx just before they entered the examination room. Patients were sedated during TEE using 25 mg pethidine and 2 mg midazolam (up to 5 mg). TEE examination was performed using a multiplane probe (Philips iE33; Philips Medical Systems, Andover, MA, USA). Mitral valve motion was categorized using Carpentier’s classification [14]. All views and measurements were stored in a picture archival and communication system (PACS, Centricity Enterprise Web V3.0, GE Healthcare, USA).

#### 2.4.2. Intraoperative TEE

Imaging and measurement of MR were started 15 min after anesthesia induction and finished before the first skin incision. During examination, excessive stimulation was avoided, and the BIS level was kept between 40 and 50. No inotropes or vasopressors were administered starting 10 min before and during the measurement period. All TEE (Philips iE33, Philips Medical Systems, Andover, MA, USA) was performed by a designated anesthesiologist who was not involved in the study and was experienced at mitral valve evaluation, having viewed 100 previous cases. All measurements were conducted more than three times and the mean value was recorded.

### 2.5. MR Data Acquisition

Preoperative TEE images were reviewed before surgery and intraoperative TEE view was optimized accordingly by the designated TEE anesthesiologist. The TEE probe was adjusted to find the jet flow most similar to the preoperative view and the MR image was zoomed. Imaging angle and the axis of flow convergence were aligned within 10 degrees before measurement. The aliasing velocity was decreased to around 40 cm·s^−1^ and the MR color flow was obtained at the end systolic phase. Maximal proximal isovelocity surface area (PISA), effective regurgitant orifice area (EROA), and regurgitation volume were obtained [15,16,17]. PISA was assumed to occur at the time of peak regurgitation volume.

### 2.6. Statistics

Our primary endpoint was mitral regurgitation volume between the two anesthetic groups. In a pilot study, we found that regurgitation volume decreased by 7.8 mL·beat^−1^ under isoflurane anesthesia with a standard deviation of 5.8 mL·beat^−1^ compared to the pre-anesthesia measurement. To detect a 20% difference in regurgitation volume between the two anesthetics, 24 patients per group were required for a power of 0.8 and an alpha of 0.05. With a 10% dropout, we planned to recruit a total of 54 patients (27 patients for each group).

Continuous variables are presented as the mean ± standard deviation (SD) or median (interquartile range) as appropriate. Categorical variables are described as count (%). The normal distribution of data was evaluated with the Shapiro–Wilk test. Confidence intervals for non-normally distributed variables were calculated using the Hodges–Lehmann estimator.

The primary outcome (regurgitation volume) between the two groups was compared using an independent *t*-test. Regurgitation volume was also compared between the two groups after adjustment for heart rate and systolic blood pressure. The distribution of MR grade pre-anesthesia and intra-anesthesia was analyzed using McNemar’s test and Pearson’s chi-square test. Other continuous variables were analyzed using an independent *t*-test or Wilcoxon rank sum test. Categorical variables were analyzed using Pearson’s chi-square test or Fisher’s exact test. Intragroup comparisons between pre- and intra-anesthesia measurements were conducted using a paired samples t-test or Wilcoxon’s signed-rank test. Statistical analyses were performed using SAS version 9.4 (SAS Institute Inc., Cary, NC, USA). A two-tailed *p*-value less than 0.05 was considered to be statistically significant.

## 3. Results

All 54 enrolled patients finished the study (*n* = 27 for each group). There were no differences in demographic or operational data between the two groups (Table 1). There were no differences in hemodynamics and the degree of pre-anesthesia MR between the two groups. Included surgeries were mitral valve repair and coronary artery bypass surgery (Table 2).

During anesthesia, systolic and mean blood pressure and left ventricular ejection fraction were lower in the isoflurane group than in the TIVA group (113 mmHg vs. 102 mmHg, *p* = 0.0228; 80 mmHg vs. 71 mmHg, *p* = 0.0304; 53% vs. 49%, *p* = 0.0072) (Table 2).

In intragroup comparisons, PISA decreased in both groups during anesthesia. However, EROA and regurgitation volume decreased only in the isoflurane group during anesthesia (Table 2).

Table 3 compares hemodynamic changes (Δ: intra-anesthesia minus pre-anesthesia) between groups. The reduction of systolic blood pressure (−1 mmHg vs. −14 mmHg, *p* = 0.0174), mean blood pressure (0 mmHg vs. −11 mmHg, *p* = 0.0194), and regurgitation volume (−0.20 mL/beat vs. −9.66 mL/beat, *p* = 0.0266) were higher in the isoflurane group than the TIVA group. Greater reduction of regurgitation volume in the isoflurane group remained consistent after adjustment of different heart rate and systolic blood pressure of the two groups (Table 4, β−coefficient=−9.18, *p* = 0.0451). The reduction of PISA and EROA were also higher in the isoflurane group than the TIVA group but did not reach statistical significance (Table 3).

The grade of pre-existing MR was also important (Table 3). Among patients with MR grade 1 or 2, the reduction in regurgitation volume was not different between the two groups (−2.24 mL·beat^−1^ vs. −2.58 mL·beat^−1^, *p =* 0.92). However, among patients with a high MR grade (grade 3 and 4), the isoflurane group had a greater reduction of regurgitation volume than the TIVA group (−0.3 beat^−1^ vs. −16.20 mL·beat^−1^, *p =* 0.0079).

**Figure 2:**
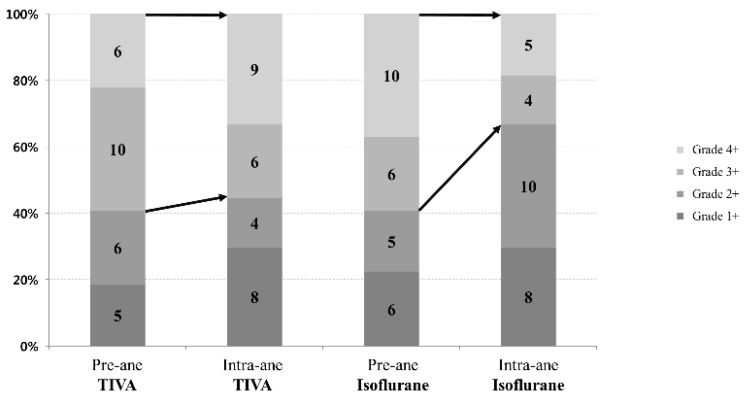
Changes in intraoperative and preoperative mitral regurgitation grade. In the TIVA group, there was no change in mitral regurgitation grade 3 and 4; however, in the isoflurane group, mitral regurgitation grade 3 and 4 decreased during anesthesia.

**Figure 3:**
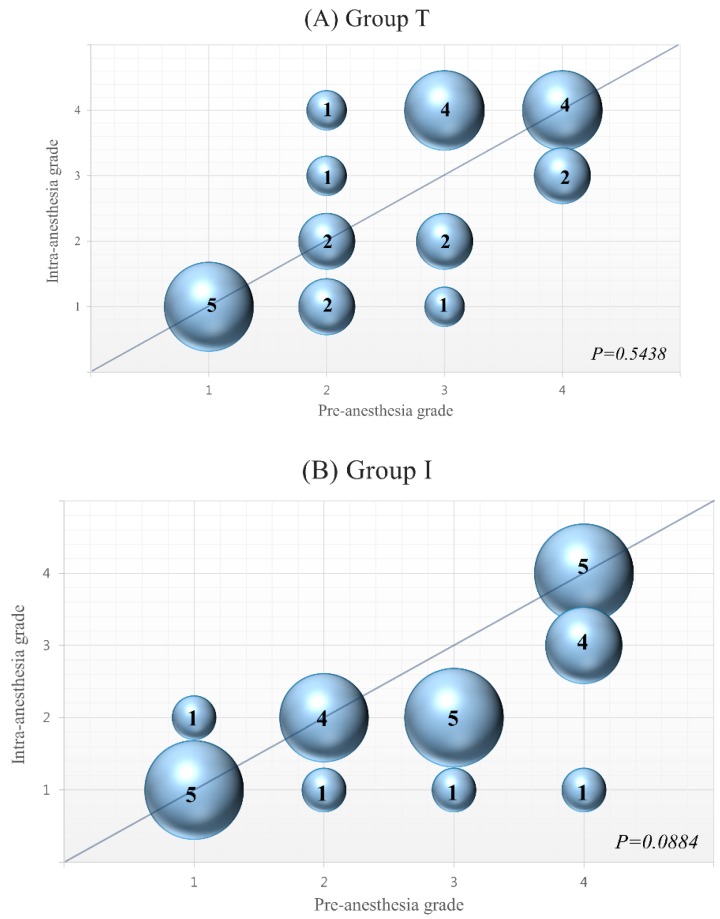
(**A**,**B**) Bubble plot. The TIVA group (**A**) maintained pre-anesthesia grade mitral regurgitation during anesthesia (*p* = 0.5438). The grade of mitral regurgitation showed a tendency of reduction during anesthesia in the isoflurane group (*p* = 0.0884) (**B**). Bubbles on the diagonal line indicate patients whose mitral regurgitation grade did not change during anesthesia. Bubbles under the diagonal line indicate patients whose MR grade decreased during anesthesia. Bubbles above the diagonal line indicate patients whose mitral regurgitation grade increased during anesthesia. X: Pre-anesthesia grade, Y: Intra-anesthesia grade.

In our study, 16 patients (59%) in each group had pre-anesthesia MR grade 3 or 4. During anesthesia, 15 patients (94%) still had MR grade 3 or 4 in the TIVA group but only 9 patients (56%) maintained MR grade 3 or 4 in the isoflurane group (*p* = 0.26) (Figure 2). In Figure 3, pre- and intra-anesthesia MR grades were plotted with a diagonal line (no change). Most patients’ data points were around the diagonal line in the TIVA group, but most of those data points were plotted under the diagonal line in the isoflurane group because intra-anesthesia MR was lower than pre-anesthesia MR.

Underling mechanisms of mitral regurgitation may also influence the regurgitation volume. Therefore, we performed a subgroup analysis according to valvular pathologies. We did not have Carpentier classification type I patients (dilated type with normal leaflet motion). Thus, the Carpentier classification type II (increased leaflet motion) and III (restrictive leaflet motion) were compared. Most hemodynamic variables failed to show difference between the TIVA and isoflurane groups including regurgitation volume in each category of Carpentier classification (Carpentier classification type II (−3 mL vs. −11 mL) and III (−4 mL vs. −8 mL), TIVA vs. isoflruane). Only blood pressures were significantly lower in the isoflurane group than in the TIVA group in Carpentier classification type III (Table 3).

## 4. Discussion

This study demonstrated that pre-existing MR may change differently according to the type of anesthetic agent. MR may be more underestimated with isoflurane anesthesia than with TIVA.

During cardiac surgery, MR is evaluated under general anesthesia. Therefore, understanding the effects of anesthetics is very important. Most previous reports agree on the reduction of MR under inhalation anesthesia. A previous study showed that MR grade decreased with isoflurane by more than 1+ in 51% of patients [18]. In a systemic review of 137 patients, intraoperative assessment under inhalation anesthesia significantly underestimated MR [19]. No reports were found on MR changes with TIVA. In the current study, the regurgitation volume did not change in the TIVA group but was reduced in the isoflurane group during anesthesia.

In our study, severe MR (grade 3 and 4) was more influenced by isoflurane than MR grade 1 and 2 (Table 3). Regurgitation volume in severe MR decreased in the isoflurane group but did not change in the TIVA group. There was no difference in low grade MR. No previous studies reported on different effect of anesthesia according to the severity of MR.

In terms of mechanisms, blood pressure and left ventricular ejection fraction were lower in the isoflurane group than in the TIVA group under the same anesthetic level (BIS 40–50). Greater reduction of regurgitation volume in the isoflurane group remained consistent after adjustment of different heart rate and blood pressure of the two groups (Table 4). These hemodynamic differences may explain the reduced MR grade in isoflurane group. Propofol mainly acts on venous smooth muscle and causes veno-dilation [20,21]. Therefore, if the preload is adequate, the cardiac output and arterial blood pressure are relatively well maintained [22]. Whereas, isoflurane reduces myofilament Ca^2+^ sensitivity with negative inotropic effects, and reduces the peak systolic left ventricular pressure by 15% at 1 MAC [23,24]. Studies comparing the degree of vasodilation and myocardial suppression between the two anesthetics are hard to find in cardiac surgery. Many confounding factors such as surgical manipulation and hemodynamic instability interrupt direct comparison of two anesthetics. Thus, animal experiments may elucidate the pure effect of anesthetics on cardiovascular system. Previous animal experiments showed that propofol maintained higher aortic pressure and increased aortic compliance and the energy transmission from the left ventricle to the arterial system [25], and showed higher arterial pressure and cardiac index, lower heart rate, and required less dopamine infusion compared to isoflurane [26].

There was also a possibility that the different types of mitral regurgitation act differently according to anesthetics. There are three types of Carpentier classifications: type I (dilated type with normal leaflet motion), II (increased leaflet motion), and III (restrictive leaflet motion). Isoflurane may reduce the degree of mitral regurgitation in Carpentier classification type I and III by myocardial depression. Propofol may mitigate the degree of mitral regurgitation in all types of Carpentier classifications if it reduces preload. We did not have type I in our study patients. Among type II and III, there were no differences in most hemodynamic variables between the TIVA and isoflurane groups probably due to lack of power (small number of patients in each sub-group). However, regurgitation volume and ejection fraction seemed to decrease more with isoflurane in both Carpentier classification type II and III, and blood pressures were significantly lower with the isoflurane in Carpentier classification type III. There are no studies yet to assess MR changes by anesthesia according to MR types. We cautiously assume that isoflurane may suppress myocardium and decrease blood pressure more than TIVA in both Carpentier classification type II and III mitral regurgitation.

Previously, anesthesiologists have administered phenylephrine or fluid during MR assessment to compensate for the decrease in pre- and after-load during inhalation anesthesia [27,28]. Shiran et al. [27], insisted that MR severity prior to inhalation anesthesia can be reproduced by maintaining systolic blood pressure at 160 mmHg by adding phenylephrine during measurement. If fluid is used, it should be administered to the point of pulmonary artery occlusion pressure of 15 mmHg or more [29]. Phenylephrine may reduce the underestimation of MR severity by inhalation anesthesia [29], but also carries a high risk of overestimation of MR [19,28]. Fluid should be challenged very carefully, especially in the weaning period, because the heart is still in the stunned state. The use of TIVA during MR assessment may reduce the need for phenylephrine or a large amount of fluid to simulate pre-anesthesia conditions.

Previous studies used the Doppler jet area color or vena contracta to measure the MR change by anesthesia [18]. Doppler jet area is abandoned for quantitative measurement due to its inaccuracy, and vena contracta is millimeter changes prone to measurement error. In the current study, we measured volume itself using PISA radius, EROA, and regurgitation volume. The PISA radius, EROA, and regurgitation volume were more decreased in the Isoflurane group than in the TIVA group, but only regurgitation volume was statistically significant.

Our study has several limitations. First, most previous MR studies were conducted by cardiologists using transthoracic echocardiography. Criteria of regurgitation volume, PISA, and EROA are not firmly established in TEE [15,30]. Second, there is the possibility of bias from different practitioners. Preoperative TEE was done by a cardiologist and intra-anesthesia TEE by an anesthesiologist. We aligned the views between the pre- and intra-anesthesia measurements as much as possible, and multiple measurements and averaged values were used. Third, we did not perform a three-dimensional assessment. Pre-operative TEE evaluation was done by two-dimensional assessment, making three-dimensional study in the operating room impossible to compare. In addition, MR quantitative analysis using three-dimensional images is limited in routine practice because of difficulty in obtaining high-quality images under surgical stimulation and prolonged time for the acquisition of the results. Forth, we used isoflurane because of its cardioprotective effect [31], but more recent anesthetics such as sevoflurane or desflurane are reported to have a similar effect with faster recovery and are recommended for further study [32].

## 5. Conclusions

Anesthesia can influence the degree of MR, especially when it is high grade. TIVA using propofol reflected the pre-existing MR state relatively well, whereas MR severity tended to decrease under isoflurane anesthesia.

## Figures and Tables

**Table 1:** Demographic data.

	TIVA (*N* = 27)	Isoflurane (*N* = 27)
Gender (Female/Male)	14/13	10/17
Age, years	61 (11)	62 (10)
Weight, kg	61 (11)	67 (13)
Height, cm	161 (8)	164 (11)
ASA PS (III/IV)	13/14	14/13
Carpentier classification (I/II/IIIa/IIIb)	0/14/9/4	0/13/9/5
**Operation**		
MVR/CABG	23/4	22/5
**Underlying disease**	16	16
Diabetes mellitus	7	7
Hypertension	8	11
Atrial fibrillation	17	16
Others	14	11
Smoking	9	6
Alcohol	12	11
Beta blocker	7	9
ACEi/ARB	7	5
Digoxin	6	2

All data are presented as mean (SD) or number. There was no difference between the two groups. Abbreviations: ASA PS, American Society of Anesthesiologists physical status classification; MVR, mitral valve repair; CABG, coronary artery bypass graft; ACEi, angiotensin converting enzyme inhibitor; ARB, angiotensin II receptor blocker; TIVA, total intravenous anesthesia; Others included chronic kidney disease, chronic obstructive pulmonary disease, cerebral infarction and thyroid disease.

**Table 2:** Hemodynamics and mitral regurgitation grade.

	Pre-Anesthesia			Intra-Anesthesia		
	TIVA (*N* = 27)	Isoflurane (*N* = 27)	*p*	TIVA (*N* = 27)	Isoflurane (*N* = 27)	*p*
HR (beat/min)	70 (54 to 81)	76 (65 to 88)	0.05	63 (59 to 86)	70 (65 to 84)	0.50
SBP (mmHg)	112 (103 to126)	112 (104 to 129)	0.62	113 (16)	102 (18)	0.023
MBP (mmHg)	80 (12)	83 (13)	0.448	80 (14)	71 (13)	0.0304
DBP (mmHg)	65 (13)	70 (13)	0.126	62 (16)	62 (13)	0.85
EF (%)	62 (59 to 64)	57 (52 to 64)	0.15	53 (52 to 60)	49 (44 to 55)	0.007
RVSP (mmHg)	43 (15)	45 (17)	0.76	39 (12)	39 (14)	0.99
RWMA	1	5	0.08	1	5	0.08
LAE/RAE	27/10	27/8	-/0.85	27/10	27/8	-/0.85
PISA (cm)	0.85 (0.17)	0.85 (0.26)	1	0.77 (0.2)	0.72(0.22)	0.39
EROA (${\mathrm{mm}}^{2}$)	34 (25 to 41)	35 (22 to 51)	0.65	25 (13 to 42)	26 (15 to 42)	0.67
RV (mL/beat)	52 (22)	54 (30)	0.76	53 (29 to 73)	36 (27 to 57)	0.25

All data are presented as mean (SD) or median (interquartile range). Abbreviations: HR, heart rate; SBP, systolic blood pressure; MBP, mean blood pressure; DBP, diastolic blood pressure; EF, ejection fraction; RVSP, right ventricular systolic pressure; RWMA: regional wall motion abnormality; LAE, left atrial enlargement; RAE, right atrial enlargement; PISA, maximal proximal isovelocity surface area; EROA, effective regurgitation orifice area; RV, regurgitation volume. Intragroup comparison, compared to pre anesthesia value: Systolic BP (Isoflurane, *p* = 0.0020); Diastolic BP (Isoflurane, *p* = 0.0155); Mean BP(TIVA, *p* < 0.0001), Ejection fraction (TIVA, *p* < 0.0001, Isoflurane, *p* < 0.0001); RVSP (TIVA, *p* = 0.0007, Isoflurane, *p* = 0.0028); PISA (TIVA, *p* = 0.0118, Isoflurane, *p* < 0.0001); EROA (Isoflurane, *p* = 0.0048); Regurgitation Volume (Isoflurane, *p* = 0.0032).

**Table 3:** Changes in hemodynamics according to mitral regurgitation (MR) grade and Carpentier classification.

**Total**	**TIVA (*N* = 27)**	**Isoflurane (*N* = 27)**	***p***
ΔHR (beat/min)	4 (−8, 13)	−3 (−11, 0)	0.10
ΔSBP (mmHg)	−1 (−8, 6)	−14 (−23, −6)	0.017
ΔMBP (mmHg)	0 (−6, 6)	−11 (−19, −4)	0.0192
ΔDBP (mmHg)	−2 (−9, 4)	−8 (−15, −2)	0.17
ΔEF (%)	−6 (−7, −4)	−7 (−9, −6)	0.18
ΔRVSP (mmHg)	−5 (−7 to −2)	−6 (−9 to −2)	0.56
ΔPISA (cm)	−0.08 (−0.14, −0.02)	−0.13 (−0.19, −0.08)	0.21
ΔEROA (${\mathrm{mm}}^{2}$)	−4.73 (−9.61, 0.15)	−9.07 (−14.64, −3.50)	0.23
ΔRV (mL/beat)	−0.20 (−6.15, 5.75)	−9.66 (−15.77, −3.56)	0.0266
**MR grade 1,2**	**TIVA (*N* = 11)**	**Isoflurane (*N* = 11)**	***p***
ΔHR (beat/min)	3 (−8, 13)	3 (−12, 19)	0.95
ΔSBP (mmHg)	4 (−9, 17)	−15 (−30, 0)	0.0410
ΔMBP (mmHg)	−1 (−13, 13)	−6 (−28, 3)	0.3091
ΔDBP (mmHg)	−5 (−18, 7)	−4 (−14, 6)	0.86
ΔEF (%)	−5 (−7, −4)	−6 (−8, −3)	0.69
ΔRVSP (mmHg)	−6 (−9, −3)	−5 (−9, −1)	0.75
ΔPISA (cm)	−0.08 (−0.17, 0.01)	−0.065 (−0.15, 0.02)	0.75
ΔEROA (${\mathrm{mm}}^{2}$)	−4.38 (−12.26, 3.50)	−0.48 (−4.78, 3.82)	0.34
ΔRV (mL/beat)	−2.24 (−7.80, 2.74)	−2.58 (−9.60, 12.20)	0.92
**MR grade 3, 4**	**TIVA (*N* = 16)**	**Isoflurane (*N* = 16)**	***p***
ΔHR (beat/min)	1 (−7, 9)	−7 (−14, 0)	0.13
ΔSBP (mmHg)	−4 (−14, 5)	−14 (−26, −2)	0.18
ΔMBP (mmHg)	−1 (11, 15)	−15 (−27, −5)	0.0373
ΔDBP (mmHg)	0 (−8, 8)	−11 (−21, −2)	0.06
ΔEF (%)	−6 (−9, −4)	−8 (−10, −6)	0.19
ΔRVSP (mmHg)	−4 (−7, 0)	−6 (−12, 0)	0.42
ΔPISA (cm)	−0.13 (−0.19, −0.04)	−0.19 (−0.24, −0.11)	0.14
ΔEROA (${\mathrm{mm}}^{2}$)	−4.98 (−11.95, 1.20)	−13.47 (−21.95, −4.98)	0.10
ΔRV (mL/beat)	−0.33 (−9.10, 8.44)	−16.20 (−24.22, −8.18) ^†^	0.0079
**Carpentier classification (** **Ⅱ)**	**TIVA (*N* = 14)**	**Isoflurane (*N* = 13)**	
ΔHR (beat/min)	1.5 (−11, 9)	−7 (−13, 3)	0.47
ΔSBP (mmHg)	−6 (−17, 1)	−9 (−20, 4)	0.94
ΔMBP (mmHg)	−4 (−13. 3)	−3 (16, 4)	0.84
ΔDBP (mmHg)	−4 (−14, 3)	−1 (−14, 6)	0.78
ΔEF (%)	−4 (−8, 0)	−10 (−11, −6)	0.12
ΔRVSP (mmHg)	−4 (−9, 0)	−5 (−10, −1)	0.69
ΔPISA (cm)	−0.11 (−0.20, 0)	−0.12 (0.18, −0.06)	0.71
ΔEROA (${\mathrm{mm}}^{2}$)	−4.75 (−12.60, 3.76)	−9.00 (−20.23, −3.24)	0.19
ΔRV (mL/beat)	−2.95 (−9.04, 5.65)	−11.00 (−14.71, −3.50)	0.10
**Carpentier classification (** **Ⅲ** **a/** **Ⅲ** **b** **)**	**TIVA (*N* = 13)**	**Isoflurane (*N* = 14)**	
ΔHR (beat/min)	6 (−4, 12)	−1.5 (−13, 12)	0.50
ΔSBP (mmHg)	2 (−4, 18)	−14 (−34, −8)	0.0021
ΔMBP (mmHg)	3 (−8, 12)	−12 (−23, −7)	0.0124
ΔDBP (mmHg)	6 (−9, 12)	−12 (−22, −3)	0.0375
ΔEF (%)	−6 (−8, −4)	−7 (−8, −4)	0.87
ΔRVSP (mmHg)	−5 (−8, −1)	−6 (−12, 1)	0.70
ΔPISA (cm)	−0.05 (−0.15, 0.03)	−0.15 (−0.23, −0.04)	0.20
ΔEROA (${\mathrm{mm}}^{2}$)	−1.60 (11.44, 1.30)	−1.90 (−13.07, 3.33)	0.97
ΔRV (mL/beat)	−3.73 (−9.22, 12.04)	−7.95 (−21.67, 1.32)	0.12

All data are presented as mean or median (95% Confidence 505 Interval). Δ, intra-anesthesia minus pre-anesthesia. Abbreviations: HR, heart rate; SBP, systolic blood pressure; MBP, mean blood pressure; DBP, diastolic blood pressure; EF, ejection fraction; RVSP, right ventricular systolic pressure; PISA, maximal proximal isovelocity surface area; EROA, effective regurgitation orifice area; RV, regurgitation volume.

**Table 4:** Hemodynamic corrected regurgitation volume.

	β-Coefficient	Standard Error	*p-*Value
ΔHR (beat/min)	0.00681	0.143	0.96
ΔSBP (mmHg)	0.023	0.118	0.85
Group T		Reference	
Group I	−9.181	4.467	0.0451

Abbreviations: HR, heart rate; SBP, systolic blood pressure; T, TIVA; I, isoflurane.
